# Distribution of Paramagnetic Fe_2_O_3_/SiO_2_–Core/Shell Nanoparticles in the Rat Lung Studied by Time-of-Flight Secondary Ion Mass Spectrometry: No Indication for Rapid Lipid Adsorption

**DOI:** 10.3390/nano8080571

**Published:** 2018-07-26

**Authors:** Lothar Veith, Antje Vennemann, Daniel Breitenstein, Carsten Engelhard, Birgit Hagenhoff, Martin Wiemann

**Affiliations:** 1 Tascon GmbH, Mendelstraße 17, 48149 Münster, Germany; lothar.veith@tascon-gmbh.de (L.V.); daniel.breitenstein@tascon-gmbh.de (D.B.); birgit.hagenhoff@tascon-gmbh.de (B.H.); 2 IBE R&D Institute for Lung Health gGmbH, Mendelstraße 11, 48149 Münster, Germany; vennemann@ibe-ms.de; 3 Department of Chemistry & Biology, University of Siegen, Adolf-Reichwein-Str. 2, 57076 Siegen, Germany; engelhard@chemie.uni-siegen.de

**Keywords:** ToF-SIMS, dark-field microscopy, lipid adsorption, amorphous silica, core/shell nanoparticles, lung tissue, protein corona formation, nanotoxicology, rat

## Abstract

Amorphous silica nanoparticles comprise a class of widely used industrial nanomaterials, which may elicit acute inflammation in the lung. These materials have a large specific surface to which components of the pulmonary micro-milieu can bind. To conduct appropriate binding studies, paramagnetic Fe_2_O_3_/SiO_2_ core/shell nanoparticles (Fe-Si-NP) may be used as an easy-to-isolate silica surrogate, if several prerequisites are fulfilled. To this end, we investigated the distribution of Fe, Si, protein and phosphatidylcholine (PC) by Time-of-Flight secondary ion mass spectrometry (ToF-SIMS) in cryo-sections from the rat lungs to which Fe-Si-NP had been administered for 30 min. Regions-of-interest were identified and analyzed with incident light and enhanced dark-field microscopy (DFM). Fe-Si-NP particles (primary particle size by electron microscopy: 10–20 nm; aggregate size by tracking analysis: 190 ± 20 nm) and agglomerates thereof were mainly attached to alveolar walls and only marginally internalized by cells such as alveolar macrophages. The localization of Fe-Si-NP by DFM was confirmed by ToF-SIMS signals from both, Fe and Si ions. With respect to an optimized signal-to-noise ratio, Fe^+^, Si^+^, CH_4_N^+^ and the PC head group (C_5_H_15_NO_4_P^+^) were the most versatile ions to detect iron, silica, protein, and PC, respectively. Largely congruent Fe^+^ and Si^+^ signals demonstrated that the silica coating of Fe-Si-NP remained stable under the conditions of the lung. PC, as a major lipid of the pulmonary surfactant, was colocalized with the protein signal alongside alveolar septa, but was not detected on Fe-Si-NP, suggesting that silica nanoparticles do not adsorb lipids of the lung surfactant under native conditions. The study shows that ToF-SIMS is a valuable technique with adequate spatial resolution to analyze nanoparticles together with organic molecules in the lung. The paramagnetic Fe-Si-NP appear well suited to study the binding of proteins to silica nanomaterials in the lung.

## 1. Introduction

Amorphous silica (SiO_2_) is among the most widely used industrial nanomaterials, which can be found in products such as tires, plastics, and lacquers. It is also used as a food additive to prevent clogging or to ensure fluidity [1]. The high abundance of SiO_2_ nanomaterials in everyday life products may lead to an increased exposure of humans to this type of nanomaterial. With respect to the workplace situation, the lung is a major route for a non-intentional uptake of SiO_2_. In contrast to crystalline silica (quartz and cristobalite), which elicit inflammation and progressive fibrosis in the lung, effects of amorphous silica appear to be confined to a transient inflammation of the lung, even at comparably high doses [2,3]. With respect to the mode of action, it is important that amorphous silica nanomaterials have a large specific surface of up to several hundred square meters per gram. It has been shown that the inflammatory effect of amorphous silica on the lung and also the cytotoxic effect on macrophages increases with surface size [4]. Of note, the inflammatory effect of colloidal SiO_2_ can be reduced by surface coating with amino or phosponate residues [5,6]. Since these surface coatings have no major influence on particle size or agglomeration behavior of the SiO_2_ nanoparticles, it is highly likely that the inflammatory effects of SiO_2_ on the lung involve an early interaction with biomolecules accessible in the lung micro-milieu. The analysis of biomolecules interacting with the surface of silica nanomaterials under in vivo conditions is, therefore, of pivotal interest.

Nanosized SiO_2_ particles, which are deposited alongside the alveolar wall, most likely bind to molecules of the lipid-rich pulmonary surfactant and/or the protein containing hypophase underneath [7]. The pulmonary surfactant covers the inner surface of lung alveoli and reduces the surface tension. It is a thin layer mainly composed of (phospho) lipids (90% by mass), with phosphatidylcholine being a major component [8]. The lung surfactant also contains specific surfactant proteins, some of which (e.g., SP-A) may bind to bacteria and, therefore, contribute to the first line of defense against invading microorganisms [9]. The lung surfactant is produced by type-2 epithelial cells and are stored in lamellar bodies. Upon secretion of lamellar bodies, the surfactant spreads and self-organizes as a monolayer whose non-polar lipids chains are directed towards the alveolar space [8]. 

Considering the spatial organization of surfactant components and the fact that nanoparticles bathed in biological fluids such as plasma rapidly acquire a protein corona [10], it is reasonable to assume that SiO_2_ nanoparticles, once deposited inside alveoli, form a corona, which, at least in vitro, can be composed of surfactant lipids and/or proteins [11]. However, because non-polar (phospho) lipid residues are facing the alveolar space, the corona of biomolecules formed around polar SiO_2_ nanomaterials under in vivo conditions has not been investigated and may be different from the corona attracted by non-polar nanoobjects such as carbon nanotubes [12]. 

The analysis of the protein corona formed around NP inside the lung is a challenging task. However, isolation of protein-laden nanoparticles (NP) would be easier, if NP could be rapidly isolated from the broncho-alveolar lavage fluid, freed from cells and non-bound components, and eventually be analyzed for their protein corona. A tempting tool for this task is paramagnetic Fe_2_O_3_/SiO_2_ core/shell nanoparticles (Fe-Si-NP). While their maghemite iron (Fe) core allows for a rapid magnetic isolation, their shell is composed of amorphous SiO_2_, to which biomolecules can bind. In this investigation, we explored commercially available paramagnetic Fe-Si-NP for the purpose of analyzing the protein corona in vivo. The main questions were: (i) How are Fe-Si-NP distributed in the lung if they are applied via intratracheal instillation? (ii) Is the SiO_2_ shell stable under the conditions of the lung? (iii) Are there any hints for a binding or accumulation of surfactant (phospho) lipids? 

To answer all these questions with a single investigation, we applied Time-of-Flight secondary ion mass spectrometry (ToF-SIMS) to sections of Fe-Si-NP-laden rat lungs. ToF-SIMS is an ideal technique to detect inorganic as well as organic materials with detection limits in the femtomole range. A lateral resolution down to 30 nm may be reached and the technique has recently been shown to unambiguously detect SiO_2_ nanomaterial in lung tissue [13]. Here, we detect Si, Fe and PC by mass spectrometry in a three-dimensional approach. Regions of interest were identified by light microscopic techniques such as enhanced dark-field microscopy, and incident light microscopy. The results of this study confirm our assumption that Fe-Si-NP are a useful tool to describe the early process of protein corona formation in the lung.

## 2. Materials and Methods 

### 2.1. Particle Characterization and Sample Preparation

The Fe-Si-NP were purchased from Kisker Biotech GmbH, Steinfurt, Germany (order No. PMSI-H.25–5). According to manufacturer, particles are composed of a maghemite Fe_2_O_3_ core and an amorphous SiO_2_ shell and had a surface area of 50 m²/g, according to the Brunauer–Emmet–Teller (BET) method. To further characterize particle size and shape by transmission electron microscopy (TEM), we dried 0.5 µL of the aqueous suspension, as used for intratracheal instillation (6 mg/mL H_2_O), onto carbon-coated copper grids. TEM analysis was carried out with a Tecnai G2 (ThermoFisher Scientific, Waltham, MA, USA). The size distribution of these aggregates (in H_2_O) was measured with a NanoSight LM10 instrument equipped with a green laser (532 nm), an Andor CCD camera, and NTA software 2.1 (Malvern Instruments GmbH, Herrenberg, Germany). 

### 2.2. Sample Preparation and Animal Experiments

Fe-Si-NP were diluted with sterile distilled H_2_O to a concentration of 6 mg/mL and ultrasonicated with a probe (VibraCell^TM^, Sonics & Materials, Danbury, CT, USA) adjusted to 50 W (20 kHz) for 10 s. This suspension was used as the final instillation fluid.

Animal experiments were conducted at the animal facility of the University Clinics of Münster, Germany, and ethically approved by LANUV (Dortmund, Germany, Accession No. 84–02.04.2022.A157). Female Wistar rats (Charles River Laboratories, Sulzfeld, Germany), weighing 200–220 g, were maintained at a 12 h lights-on lights-off cycle; food and water were provided ad libitum. To administer Fe-Si-NP into the lung, animals were briefly anaesthetized with isoflurane and intratracheally instilled with 500 µL of instillation fluid containing 3 mg (*w/v*) Fe-Si-NP using a Penn Century Microsprayer. After 30 min, rats were deeply anaesthetized with ketamine/xylazine and bled via the descending aorta. A cannula was inserted into the trachea and the lung was lavaged five times with 5 mL 0.9% NaCl, to obtain lavage fluid for an accompanying investigation (data not included). Thereafter, the lung was inflated with 5 mL Cryomatrix (Thermo Shandon Ltd., Runcorn, UK), resected, snap frozen in liquid nitrogen, and stored at −80 °C for histological studies. Transverse sections were cut from the hilar region of the left lung with a cryo-microtome (HM 500, MICROM International GmbH, Walldorf, Germany). For ToF-SIMS analysis, 7 µm thick sections were dried onto ITO-coated glass slides (Sigma Aldrich, Germany) and stored at −20 °C to investigate Fe-Si-NP. Of note, the lavage procedure as carried out here leaves a considerable number of e.g., cells (>70%) within the lung parenchyma [14]. The same is true for particles attached to the alveolar walls as shown below.

### 2.3. Enhanced Dark-Field Microscopy

Enhanced dark-field microscopy (DFM) makes use of highly intense white light and allows to visualize particles and NPs in cells and tissues with strong light scattering properties down to a size of 20–30 nm. To apply the technique, air-dried cryo-sections were fixed in formaldehyde for 10 min, washed in phosphate buffer and cover-slipped using an aqueous mounting medium (Shandon Immu-Mount, Thermo Fisher Scientific, Bremen, Germany). Sections were viewed with an upright microscope (Olympus BX51) and digital bright-field images were captured using a RETIGA 2000R camera (Q Imaging, Surrey, BC, Canada). Areas of interest were then imaged with a DFM microscope, equipped with a 40-fold UPlanApo Oil Iris objective (Olympus, Hamburg, Germany), and a CytoViva enhanced dark-field condenser. All DFM components were purchased from CytoViva, Inc. (Auburn, AL, USA). 

### 2.4. Topographic Analysis

DFM imaged slides were cautiously immersed and rinsed in H_2_O to remove the coverslip and embedding medium, respectively. The sections were air-dried again and analyzed with an optical profiler instrument (PLu neox, Sensofar-Tech, Barcelona, Spain). A 50-fold EPI-objective (numerical aperture: 0.80) was used to acquire images with a resolution of 768 × 576 pixels for the selected area. The corresponding dimensions of a single pixel are 330 × 330 µm², which corresponds to a pixel density of 3.03 pixels/mm. Up to 33 consecutive images were acquired at a focal distance of 0.2 µm by an automated routine. The image stack was converted into a single sharp image with ImageJ software using the extended depth of field plugin [15] or used to generate a topographic image using SensoScan software (Version 3.5.2). 

### 2.5. ToF-SIMS Analysis

To confirm the chemical identity of the nanoparticles and their Fe/Si core/shell structure a droplet of the aqueous Fe-Si-NP nanoparticle suspension was dried onto aluminum foil. ToF-SIMS spectra were acquired in the spectrometry mode at a TOF.SIMS^5^ (IONTOF, Münster, Germany) for the pre-characterization of the nanoparticles. The raster size was 120 × 120 µm² at 128 × 128 pixels. 25 keV Bi_3_ ions were applied at an ion dose of 1.5 × 10^8^ ions and a cycle time of 200 µs. The corresponding mass range reached from 1 Da up to 1650 Da.

Measurements using O_2_ sputtering were performed using Bi_3_ at 25 keV with an ion dose of about 2.2 × 10^10^ ions applied to an analysis field-of-view of 300 × 300 µm² with a pixel raster of 512 × 512 pixels. O_2_^+^ was used as a sputter ion at an energy of 1 keV. A total sputter dose of 8.0 × 10^14^ ions was applied to a sputter raster size of 700 × 700 µm². The delayed extraction mode in combination with non-interlaced sputtering was used at a cycle time of 80 µs resulting in a mass range from 1 Da to 550 Da. Three replicate analyses were conducted to assure the validity of the obtained data.

### 2.6. Correlation Analysis

Image processing was carried out with the FIJI distribution for ImageJ (National Institute of Health, Bethesda, MD, USA) [16,17]. The colocalization threshold plugin was used for the colocalization analysis [18]. The image registration was executed with the Turboreg [19] plugin (Biomedical Imaging Group, EPFL Lausanne, Switzerland). 

## 3. Results and Discussion

### 3.1. Electron Microscopic and ToF-SIMS Characterization of the Nanoparticles 

**Figure 1:**
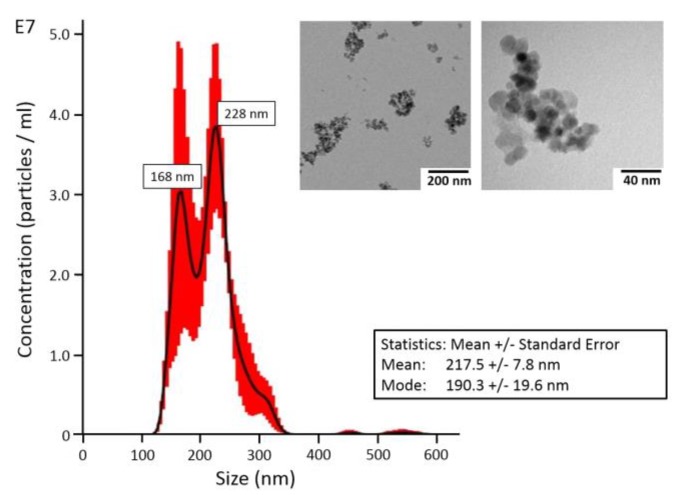
Size distribution of Fe-Si nanoparticles (Fe-Si-NP) in aqueous suspension as used for intratracheal instillation. Measurements were carried out by NanoSight optical tracking analysis. The inset shows electron microscopic images of Fe-Si-NP as typically found in the instilled suspension.

TEM analysis showed that Fe-Si-NP consisted of nanoparticles with a size of ca. 10–20 nm, which mainly formed larger aggregates (Figure 1, inset). Fe-Si-NP dispersed in H_2_O showed two maxima at 168 nm and 228 nm, as analyzed with optical tracking analysis; mean and mode values amounted to 217.5 ± 7.8 nm and 190.3 ± 19.6 nm, respectively (Figure 1).

**Figure 2:**
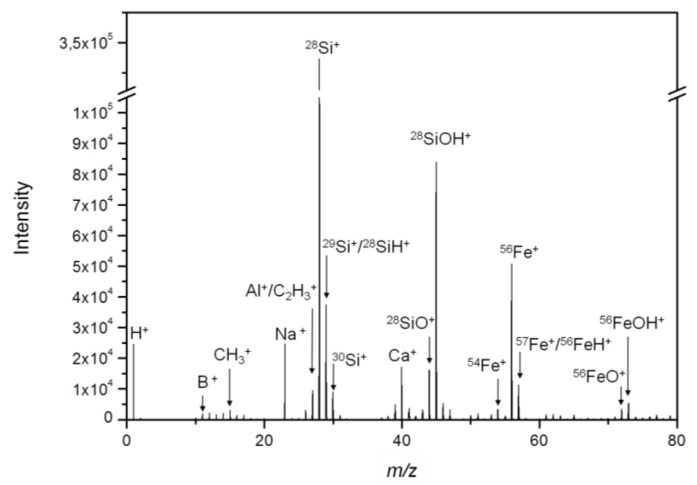
Mass spectrum of the Fe-Si-NP with a Fe/Si core/shell structure. Particles were deposited on an aluminum substrate and analyzed by ToF-SIMS. The particle components Si_x_O_y_H_z_^+^ and Fe_x_O_y_H_z_^+^ and their respective isotopes are detected along with hydrocarbons (CH_3_^+^ and C_2_H_3_^+^), substrate components (Al^+^), and other residues possibly linked to the production process (B^+^, Na^+^, and Ca^+^).

The mass spectrum, as revealed by ToF-SIMS, shows several prominent Si- and Fe-containing species (Figure 2), from which ^28^Si^+^, ^30^Si^+^, ^54^Fe^+^, ^56^Fe^+^ and ^56^FeOH^+^ were selected as relevant secondary ions for the detection of Fe-Si-NP in lung tissue sections. Besides the often detected hydrocarbons [20], B^+^, Na^+^ and Ca^+^ were detected with notable intensities and these substances are most likely associated with the particle production process.

While the findings illustrate the need for an extensive pre-characterization of nanoparticles before their use in toxicology studies, they also indicate several characteristic secondary ions (^28^Si^+^, ^30^Si^+^, ^54^Fe^+^, ^56^Fe^+^, and ^56^FeOH^+^) that can be used for the detection of the nanoparticles in the tissues.

The presence of Fe upon primary ion bombardment suggests that the ToF-SIMS information depth might be greater than the thickness of the Si shell. This finding can be explained by the highly energetic primary ion beam, which induces significant amounts of damage to nanoparticles and sometimes may even disrupt the particle. Sandoval et al. found an increased secondary ion yield for nanoparticles compared to bulk materials [21]. Increases in the secondary ion yield are always to be expected from nanoparticles depending on their sizes, because they have a larger surface area than bulk materials. However, the performance of the collision cascade is affected, if the size of the particles is in the same order of magnitude as the collision cascade. Obviously, this can also lead to increased intermixing of the particle components. Yang et al. observed a deformation of nanoparticles upon ion bombardment [22], which could also explain the simultaneous detection of core and shell components observed here for Fe-Si-NP. In any case, the simultaneous detection of the Si and Fe signals without the need for sputtering was beneficial for the detection of the core/shell particles in the tissue.

### 3.2. Inspection of Fe-Si-NP-Laden Lung Tissue by Light Microscopy

Light microscopic techniques are increasingly used to detect nanoparticles in tissue sections [23]. Especially enhanced dark-field microscopy can detect light scattering particles down to a size of 20–30 nm [24]. Furthermore, metallic or metal oxidic nanoparticles often have plasmonic, reflective and diffractive properties, which allow for their differentiation against translucent tissue [25], or, in ideal cases, for identification via hyperspectral imaging [26]. Here, bright-field microscopy combined with DFM was used to localize Fe-Si-NP in the lung tissue prior to ToF-SIMS analysis.

**Figure 3:**
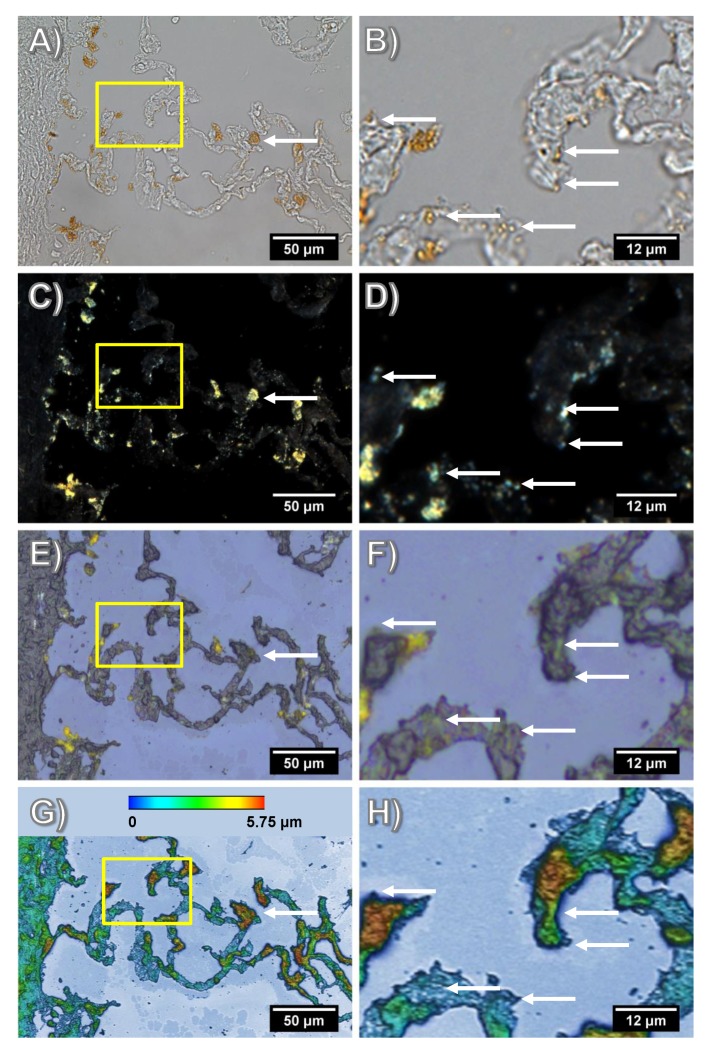
Light microscopic analyses of a Fe-Si-NP-laden cryo-section from rat lung. Overview (**A**,**C**,**E**,**G**) and detail (**B**,**D**,**F**,**H**) of the boxed area shown on the left. (**A**–**D**) Routine images of the coverslipped section taken with bright-field optics (**A**,**B**) and DFM (**C**,**D**); Fe-Si-NP agglomerates appear as brownish patches at alveolar septa or in macrophages. With DFM, additional small light-scattering particles are seen alongside alveolar septa. (**E**–**H**) Same tissue area after removal of the coverslip, rinsing and drying. (**E**,**F**) Microscopic image of the dry section without coverslip, taken with a reflective light microscope. Prominent Fe-Si-NP agglomerates are still identifiable as yellow patches. (**G**,**H**) Optical profilometry analysis showing the relief structure of the dry lung section as subjected to ToF-SIMS; the topographic map reveals an amplitude of several micrometers. Arrows in (**A**,**C**,**E**,**G**) point to a particle-laden macrophage (**A**,**C**), which became lost in the course of tissue preparation. Similarly, arrows in (**B**,**D**,**F**,**H**) point to small Fe-Si-NP (seen in (**B**,**D**)), which were absent after drying.

Although the microscopic detection of SiO_2_ nanomaterials in tissue sections is hampered by low optical contrast, the iron core of Fe-Si-NP caused a “rust”-like color facilitating the identification of larger agglomerates in bright-field images (Figure 3A–F). DFM confirmed this distribution but additionally revealed smaller yellow or blue dots in, or attached to, the hardly light scattering tissue structure (Figure 3C,D). Although the detection of nanoparticles in tissues by DFM is generally a straightforward approach, it is obvious that any light scattering inhomogeneity might lead to misinterpretation. 

Bright-field microscopy and DFM demand that the tissue section is immersed in ringer solution, coverslipped and viewed with oil-immersion lenses. As ToF-SIMS analysis is carried out under vacuum conditions, the coverslip has to be cautiously removed and the sections needs to be dried. Mild shearing forces and washing steps are indispensable during this procedure and may influence the signals gathered from particles in the subsequent ToF-SIMS analysis. To show these effects, reflective light microscopic images were taken at the same position of the dried section. 

Reflective light microscopic images have a lower optical resolution but are highly useful to visualize the structure of a section prepared for ToF-SIMS analysis. Here, it is shown that numerous large yellow spots (up to 15 µm) previously seen in the bright-field or DFM image were still in place, whereas others structures, which were interpreted as Fe-Si-NP filled macrophages, were no longer visible (Figure 3E,F; white arrows). Similarly, smaller particles (<3 µm), which are marked by arrows in the magnified bright-field and DFM images (Figure 3B,D), were not seen on reflective light microscopy images. 

As ToF-SIMS analysis makes use of sputtering and ion bombardment, which is applied under a certain angle, the relief structure of the dried tissue section will unavoidably influence image resolution. Figure 3G,H shows the relief of the dried tissue on the indium tin oxide (ITO)-coated glass substrate, as analyzed by profilometry. The height of the formalin-fixed and afterwards dried alveolar septa on the substrate ranged 2.5–5.25 µm, which was 36–75% of the initial thickness of the cryo-section (7 µm) and may be explained by evaporation of H_2_O prior to immersion fixation. Although the nature of the highest peaks remains unknown, these sites do not contain agglomerated Fe-Si-NP, which were mainly found in areas ranging in height from 2–3.5 µm. 

In summary, the combination of several microscopic methods showed that Fe-Si-NP were distributed alongside the alveolar wall or were contained in macrophage-like structures. The successive observation by DFM and ToF-SIMS may lead to a loss of Fe-Si-NP containing structures and this has to be considered when both images are compared (see below). 

### 3.3. ToF-SIMS Detection of Nanoparticles in Tissue—Localization

In the next step, we tested the intactness of the Fe/Si core/shell in the tissue using both the Fe- and Si-signal distributions in the ToF-SIMS analysis. A sputter erosion with 1 keV O_2_^+^ ions was necessary to analyze the complete depth of the sample section.

**Figure 4:**
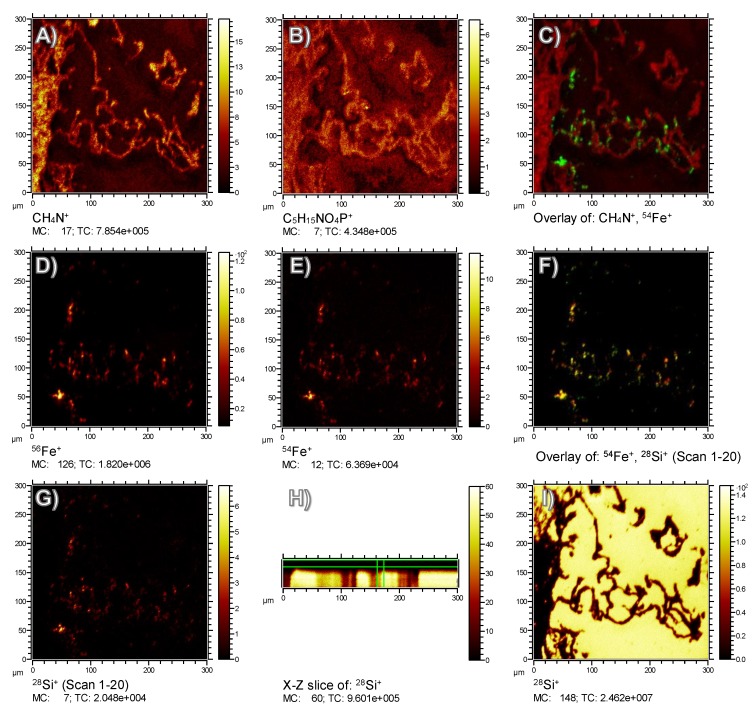
ToF-SIMS signal distributions of organic and inorganic components in a Fe/Si core/shell nanoparticle-containing lung tissue section 30 min post intratracheal instillation of particles. (**A**) CH_4_N^+^ indicative of proteins to show tissue distribution and lung structure. (**B**) C_5_H_15_NPO_4_^+^ is the phosphocholine (PC) head group fragment showing the distribution of lipids of lung tissue and surfactant; PC signal seen in the alveolar space is interpreted as to be artificially dislocated during filling of the lungs with cryomatrix. (**C**) Overlay of ^54^Fe^+^ (green) and CH_4_N^+^ (red) signal showing Fe-Si-NP distribution in lung tissue. (**D**) ^56^Fe^+^, and (**E**) ^54^Fe^+^ distributions of Fe-Si-NP particles. Small and large agglomerates appear alongside the alveolar walls. (**F**) The overlay of ^54^Fe^+^ (**D**, red) and ^28^Si^+^ (**G**, green) reveals the colocalization of both particle-related signals. (**G**) ToF-SIMS signal distribution of Si species from the topmost layers as indicated by the horizontal green lines in the X-Y slice of the ToF-SIMS dataset in (**H**). (**I**) ^28^Si^+^ signal distribution integrated for the complete depth including the indium tin oxide-coated glass slide.

The tissue structure of the lung with all its alveolar septa, as shown in Figure 3, was properly reflected by the CH_4_N^+^ signal, which is a typical fragment of amino acids and, thus, indicates the position of fixed proteins (Figure 4A). Due to the relatively harsh sputtering conditions of 1 keV O_2_^+^ ions, only few organic molecular species were preserved. Nevertheless, the lateral distribution of the signal at *m*/*z* 184.07 could be summed up over the whole depth of the analysis. This fragment is indicative of the phosphocholine head group C_5_H_15_NPO_4_^+^ and most likely attributable to phospholipids previously located in cell membranes and/or in the pulmonary surfactant. A minor fraction of the signal was found in alveolar spaces (Figure 4B), suggesting that it was transferred onto the substrate during the preparation process. 

With respect to Fe, several relevant signals (besides the main isotopes ^54^Fe^+^ and ^56^Fe^+^) were identified in the spectrum of both, the isolated and the lung-incorporated Fe-Si-NP (Figure 2). Of note, further iron-related species with the same lateral distribution were found, e.g., ^56^FeOH^+^, ^54^FeOH^+^, and ^56^Fe_2_O^+^, proving that the signals were correctly assigned to all Fe species. In any case, the lateral signal distribution of the main Fe isotopes ^54^Fe^+^ and ^56^Fe^+^ (Figure 4D,E) was largely congruent with the distribution of Fe-Si-NP in the tissue (Figure 3C,E). This is particularly obvious for some larger areas with diameters of up to 15 µm (Figure 4D,E), but also for numerous smaller and less intense Fe patches. As most of these areas have a diameter larger than 200 nm, they most likely represent Fe-Si-NP agglomerates. 

Colocalization with the CH_4_N^+^ signal (Figure 4C) shows that the majority of Fe signals (green) in the range of 3–10 µm was localized alongside the alveolar septa (red), which is in accordance with the microscopic results of Figure 3 and was expected due to the short incubation time of only 30 min. Some of the smaller Fe-signals (green) were colocalized with the epithelium and might be interpreted as to be incorporated by the epithelial cell. However, due to the preparation steps of the lung prior to fixation (filling with cryomatrix, freezing, sectioning, and drying) and to the relief structure of the tissue (Figure 3G), a precise cellular localization of particles cannot be made by light microscopy or ToF-SIMS. A study by Shon et al. carried out on air-dried unfixed macrophages laden with dimercaptosuccinic acid-coated Fe_3_O_4_ nanoparticles succeeded in localizing particles in the cytoplasm [27]. Phosphatidylcholine components in that study were detected as specific C_4_H_7_^+^ ions only.

### 3.4. Colocalization of Fe and Si in Lung Tissue by ToF-SIMS

With respect to the simultaneous identification of Fe and Si, isobaric interferences may occur due to the similarity of the theoretical mass of ^56^Fe^+^ (*m*/*z* 55.934939) and ^28^Si_2_^+^ (*m*/*z* 55.953854). This hampers a reliable distinction by mass, and only an improvement of mass resolution from the current R = 1500 to R > 3000 could solve this problem. However, the ^54^Fe^+^ signal, although relatively weak, is not influenced by any Si cluster and, therefore, allows analyzing the Fe distribution unbiased from Si signals. Thus, adopting ^54^Fe^+^ as a reference, it turned out that ^56^Fe^+^ signals virtually showed the same distribution as did ^54^Fe^+^. In addition, the ^56^Fe^+^ signal was hardly influenced by ^28^Si_2_^+^ from any non-particle source and showed a low background intensity in tissue-free areas (Figure 4D,G). Due to the obvious correlation of the signal distributions for the ^54^Fe^+^ and ^56^Fe^+^ isotopes, we decided to use the ^56^Fe^+^ ion to measure the distribution of Fe in lung sections. 

To identify Si on the paramagnetic Fe core, any Si signal from the ITO-covered glass substrate had to be avoided. Therefore, the ^28^Si signal was reconstructed from only the first 20 sputter/analysis cycles (i.e., scans). The signal distribution obtained by this was highly similar to the Fe distributions (Figure 4G), indicating that the ITO layer had successfully shielded the underlying glass substrate against the analysis beam. In contrast, if the signal was summed up over all 81 cycles (compare X–Z slice in Figure 4H), the sputter cycles had eroded the ITO layer and the apparent ^28^Si^+^ distribution reflected the underlying substrate in tissue-free regions (Figure 4I). Based on selected data evaluation and an isotopic signal distribution, the presence of Si on nanoparticles could be confirmed even in the positive ion polarity upon oxygen sputtering, as opposed to earlier studies using Cs sputtering and the negative ion polarity [13]. Consequently, the identification by ToF-SIMS of pure, unlabeled SiO_2_ nanoparticles in tissue sections should be possible with this protocol, provided that any contamination, e.g., with the widespread polysiloxanes, can be ruled out.

Another option to identify correlating distributions of Si and Fe species is the comparison of Fe isotope distributions with ^30^Si, as oxide clusters of this ion cannot form signals with the same mass-to-charge-ratio as the Fe isotopes in the observed mass range. However, as ^30^Si is also a part of the ITO shielded substrate, the same limitations as for ^28^Si apply. Of note, the reconstruction of the less abundant ^30^Si^+^ isotope ruled out that the ^28^Si^+^ signal merely originated from ^56^Fe^+^ (*m*/*z* 27.967470), which would partially interfere with the ^28^Si^+^ (*m*/*z* 27.976927), since both, ^28^Si and ^30^Si show the same signal distributions (not shown here).

Based on the comparison of Figure 4D,E (for Fe) and Figure 4G (for Si), the most important result is that the ^28^Si^+^ distribution derived from the first 20 sputter/analysis cycles was properly colocalized with the Fe distributions, as is shown by the numerous yellow spots in Figure 4F, which result from red ^54^Fe pixels overlaid with green ^28^Si pixels. This demonstrates that the Si shell of the paramagnetic Fe particles is stable under in vivo conditions. 

### 3.5. Correlation and Comparison of Dark-Field Microscopy and ToF-SIMS

**Figure 5:**
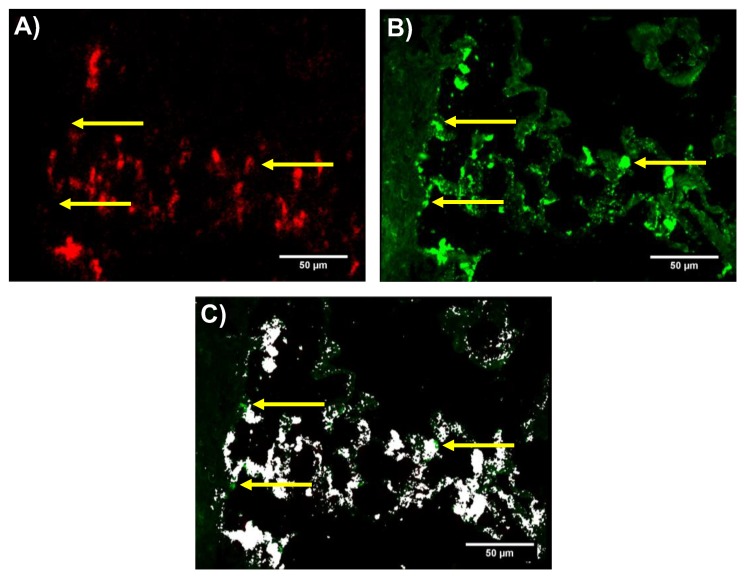
Comparison of ToF-SIMS ^54^Fe^+^ signal distribution and dark-field microscopy (DFM) images. Image taken from Figure 3. (**A**) ^54^Fe^+^ ToF-SIMS signal (red); (**B**) DFM signal (green) taken before washing and ToF-SIMS analysis; and (**C**) Colocalization of (**A**,**B**). A high degree of correlation is evident (white overlay). The Pearson correlation coefficient for this image was 0.541. Arrows point to green spots (**B**) which were detected by DFM, but not by ToF-SIMS.

Enhanced dark-field microscopy (DFM) detects NP down to a size of 15 nm [28] and was, therefore, used as a sensitive tool to visualize the Fe-Si-NP in tissue sections prior to ToF-SIMS analysis. Overall, the ToF-SIMS ^54^Fe^+^ signal and the DFM signal showed a high degree of similarity (Figure 5A,B). Colocalization analysis of both signals using Pearson’s correlation coefficient confirmed this high degree of co-localization. As this coefficient ranges from −1 (perfect anti-correlation) to +1 (perfect correlation), a value of 0.541 confirmed that a considerable number of pixels (^54^Fe^+^ positive by ToF-SIMS) correlate with the bright spots in the DFM image (Figure 5C). Of note, this result strongly suggests that ToF-SIMS had not detected false positive signals. 

However, the correlation was not perfect and differences were observed for some larger dots (marked in Figure 5), most likely corresponding to particle-laden macrophages, but also for smaller particles seen in DFM. As outlined above, especially inappropriately fixed material may have become lost during preparation. 

Other reasons may also account for such differences: Fe-Si-NP might not have been detected by ToF-SIMS due to insufficient sample erosion (false negative ToF-SIMS signals). However, as the sputtering with O_2_^+^ and subsequent analysis were extended into the depth of the substrate, it appears unlikely that the soft lung tissue shielded Fe-Si-NP and prevented the accumulation of supra-threshold signal intensities in the ToF-SIMS analysis. Rather, the accessible mass (i.e., particle size and/or concentration) was too small, and a low signal did not reach the limit of detection. This appears unlikely for most of the Fe-Si-NP aggregates larger than 100 nm but may account for the 10–50 nm fraction of Fe-Si-NP, which is visible by TEM (Figure 1) and, at least in part, should be visible by DFM.

Finally, unlike the large brown agglomerates (Figure 3A,B), small objects can scatter light as well and may have been erroneously designated as Fe-Si-NP by DFM. Hyperspectral microscopy may be used to further identify such questionable structures.

In summary, the ion distribution measured by ToF-SIMS explains most of the DFM signals, thereby serving as a validation technique. However, DFM images contained several signals without any ToF-SIMS equivalent. These DFM signals were most likely caused by salt residues or tissue material and indicate that it is necessary to confirm the chemical identity of nanoparticles in DFM imaging studies. 

### 3.6. Lack of Colocalization of Phophatidylcholine with Fe-Si-NP and General Impact of Findings

In this study, most of the nanomaterial was found closely related to the alveolar septa by both optical and mass spectrometric techniques (Figure 3 and Figure 4G). In addition, a partial uptake into macrophages was observed despite the relatively short incubation time of 30 min. The obviously persistent macrophage activity suggests that the intratracheal instillation of H_2_O, which was used to favor the dispersion of the polar NP, has no negative impact on the micro-milieu of the lung. Nevertheless, larger agglomerates of Fe-Si-NP with diameters of several micrometers were found in the lung. These agglomerates may have formed upon contact with the lung lining fluid and/or during lymphatic retrieval of the aqueous suspension fluid. As such, an agglomeration of nanoparticles in lung instillation studies is common [29], although, e.g., citrate-stabilized gold nanoparticles do not agglomerate under these conditions [30]. 

In any case, it appears plausible that Fe-Si-NP, which had a size of less than 200 nm (see Figure 1) when they were administered to the lung, may have come into direct contact with several well organized layers of biomolecules such as the lung surfactant, the hypophase, and/or the membrane of (epithelial) cells. It appears conceivable that this sequential arrangement of biomolecules and ions in the lung orchestrates the formation of the protein corona as well as agglomeration of NP. A protein corona, which can be artificially generated with different preparations of lung surfactant [11] may, therefore, differ from the native corona. 

**Figure 6:**
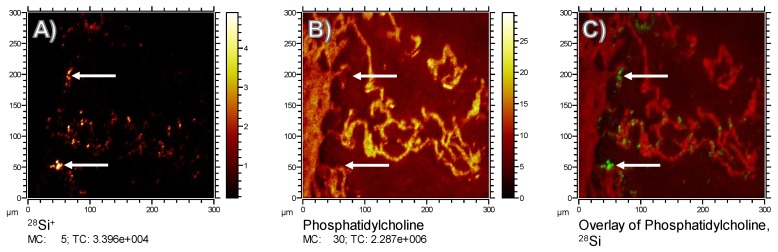
Comparison of the Si and phosphocholine distribution in a cryo-section of the rat lung 30 min post administration of Fe-Si-NP. All images contain information of the uppermost 20 scans only. (**A**) ^28^Si^+^; (**B**) C_5_H_15_NPO_4_^+^, which is the 184.07 fragment of the phosphocholine (PC) head group showing the distribution phosphatidylcholine; and (**C**) the overlay of (**A**,**B**). Arrows in all images point to the position of larger agglomerates of Fe-Si-NP; note that in (**B**) PC is not detected at the indicated sites.

To monitor the putative binding of (phosho) lipids to silica nanoparticles in the lung, we compared the *m*/*z* 184.07 signal (representing phosphatidylcholine as the main constituent of pulmonary surfactant and cell membranes), with the distribution of Si, which forms the outer shell of the Fe-Si-NP: While the *m*/*z* 184.07 signal delineated the alveolar septal structure, and was also found on the ITO substrate at lower intensities, volumes occupied by the Si signal in the first 20 scans were completely free of PC (Figure 6, arrows). Although we are aware that the sensitivity of analytical methods can be improved, e.g., by well-adapted sputtering methods, we conclude that there is no major accumulation of phospholipids on the polar surface of Si nanoparticles. We furthermore suggest that phospholipids, as constituents of the lung surfactant, are not involved in the formation of particle agglomerates.

This finding seems to be in contrast to a recent investigation on lipid binding to SiO_2_, ZrO_2_, or AlOOH in vitro [11]. Using two different preparations of pulmonary surfactant, it was found that polar nanoparticles bind indeed to phospholipids, if the mixture (Curoserve^®^) contained surfactant protein A. However, the role of surfactant proteins for an in-situ opsonization of nanoparticles is still not known. As mentioned above, the incubation of nanoparticles in artificial or isolated pulmonary surfactant may be profoundly different from binding conditions in the lung. While in the lung the air–liquid interface carries non-polar lipid residues facing the gas phase, lipids in isolated lung surfactant may form micelles with their polar residues directed outwards. 

The current results provide, for the first time, new and more realistic insights in the formation of the lipid/protein corona around silica nanoparticles under native conditions of the lung. Findings may be representative for pulverulent silica nanomaterials, unless they are coated, e.g., by hydrophobic molecules. These findings have been achieved with high-resolution ToF-SIMS, a state-of-the-art bio-imaging method, which is universally applicable and circumvents the need to isolate particles from tissues. Future developments of the technique such as an ultra-high mass resolution detector are in sight, which may further increase the performance of the method [31]. This will allow us to obtain more detailed analyses of molecules bound to NP under in vivo conditions. 

### 3.7. Methodical Considerations

It is well known that any type of tissue preparation may have an influence on bio-imaging results. As will be described below (3.2–3.4), tissue sections analyzed by ToF-SIMS were taken from lavaged lungs, which were filled with a cryomatrix and immediately snap-frozen in liquid nitrogen. Although filling with a cryomatrix preserves the tissue architecture of the lung, the compound does not fix subcellular structures, such that a reliable distinction between cell-attached and intracellular NP cannot be made. Furthermore, sections were cut, dried onto ITO slides, stored in a frozen state, and eventually fixed with formalin on the slide. We are aware that this procedure cannot prevent a partial loss of NP, especially if they are not in contact with fixable tissue components. However, numerous particles and agglomerates proved to be firmly attached, because even the withdrawal of the coverslip prior to ToF-SIMS analysis left the majority of particles in place, as shown in Figure 5. 

There may also be a partial loss or wash-out of lipids, as these molecules are not fixable with formalin. However, neither ethanol nor other organic solvents were used, such that PC molecules remained highly abundant in all tissue structures. Nevertheless, there was a low background signal of PC in alveolar areas, which may be interpreted as an artificial displacement of PC. However, as levels of PC were low in the vicinity of the alveolar septa (Figure 6B), we assume that PC found within alveoli was not displaced from cellular structures. Rather, it may reflect a mixture of lung surfactant and liquid cryomatrix which dried onto the ITO carrier. Considering all these facts, we cannot completely rule out that the lack of PC on Fe-Si-NP was partially artificial, but the complete absence of PC over all Si signals (see Figure 6C) makes this interpretation highly unlikely. 

Finally, the lack of PC on Fe-Si-NP might also hint at an inappropriate sensitivity of the ToF-SIMS method. However, in a previous investigation on the composition of Langmuir–Blodgett films, PC and other phospholipids were successfully identified in a lung surfactant-like monolayer [32]. 

Thus, although some artificial displacement of particles and/or a loss of PC cannot completely be ruled out, we suggest that the absence of PC on the silica surface of the Fe-Si-NP in the lung, as measured by the highly sensitive ToF-SIMS method, is not an artifact and, therefore, of biological significance.

## 4. Conclusions

In this study on paramagnetic silica nanoparticles (Fe-Si-NP) administered to the lung via intratracheal instillation for 30 min, we show that particles were attached to alveolar walls and only marginally internalized by cells such as alveolar macrophages. Due to the successful colocalization of Fe and Si signals by ToF-SIMS analysis of lung cryo-sections, we conclude that the core/shell structure of the Fe-Si-NP remains intact under these conditions, such that Fe-Si-NP may now be used to study the early protein adsorption to a nanosized silica surface in the lung. Finally, the analysis of the phosphocholine head group, as a representative of phosphatidylcholine (PC), which is a major lipid of the pulmonary surfactant, revealed no augmented PC binding to Fe-Si-NP. This is an important finding as previous in vitro studies suggested that, e.g., surfactant protein A could mediate the binding of phospholipids to the polar silica surface. In this respect, ToF-SIMS proved to be a valuable technique with high spatial resolution and appropriate limits of detection, which made it possible to analyze nanoparticles together with bound organic molecules within their cellular environment.
